# Pantoea in Peritoneal Dialysis: A Rare Cause of Peritonitis

**DOI:** 10.7759/cureus.26878

**Published:** 2022-07-15

**Authors:** Catarina Mateus, Ana Rita Martins, Cristina Toscano, Patrícia Matias, Patrícia Branco

**Affiliations:** 1 Nephrology, Centro Hospitalar Lisboa Ocidental - Hospital de Santa Cruz, Lisbon, PRT; 2 Microbiology, Centro Hospitalar Lisboa Ocidental - Hospital Egas Moniz, Lisbon, PRT

**Keywords:** pantoea agglomerans, erwinia, peritoneal dialysis, pantoea, peritonitis

## Abstract

Peritonitis is the most common complication of peritoneal dialysis (PD) and an important cause of PD failure. There are numerous etiological agents, mostly bacteria. *Pantoea spp* is a rare cause of peritonitis.

We describe three cases of *Pantoea* peritonitis in three PD patients. Previous reports have identified risk factors such as close contact with plants and animals. We review the typical clinical presentation and prognosis. It is fulcral to teach patients about the risks regarding proximity to plants and animals to prevent this type of infection.

## Introduction

*Pantoea* spp is a gram-negative bacterium that belongs to the *Erwiniaceae* family. It is defined as ubiquitous and is found in plants, flowers (such as roses), fruits, and vegetables and also in the oral cavity and feces of humans and animals. Its pathogenicity for humans was reported back in the 1970s [1]. Since then, it has been described as a causative agent of bacteremia, soft tissue, bone, and joint infection in the pediatric population and the cause of infection due to contamination of medical products, blood, and intravenous drugs or fluids [2-5]. It was first linked to peritoneal dialysis-associated peritonitis (PD peritonitis) in 2005, with a case report of a two-year-old patient with polymicrobial peritonitis [6]. As far as we know, only 11 other cases have been reported; therefore, *Pantoea* infection is a rare infection in this population.

This article reports three cases of PD peritonitis due to *Pantoea* spp. We also believe that *Pantoea* spp might be an underdiagnosed agent of infection in PD patients due to difficulties in the diagnosis.

## Case presentation

Case I

A 63-year-old Caucasian man with chronic kidney disease (CKD) stage 5 due to hypertension and horseshoe kidney with calcium oxalate calculi was started on peritoneal dialysis (PD). One year after starting PD, he complained about abdominal pain for two days. He had been gardening and had abrasive wounds in the forearms caused by rose thorns. The patient remained apyretic and hemodynamically stable, with abdominal tenderness and cloudy drainage fluid. The diagnosis of PD peritonitis was straightforward. The Tenckhoff catheter exit site had no inflammatory signs. Laboratory blood tests revealed a white cell count (WCC) of 6.0x10^9^ with 69% neutrophils and elevated C-reactive protein (CRP) level of 12.8 mg/dL. Peritoneal cell count (PCC) was 6.720 cells/µL with 74% of neutrophils. He was treated with intraperitoneal cefazolin and ceftazidime as an outpatient. Gram stain examination revealed Gram-negative bacilli in the ascitic fluid, and cultures identified *Pantoea* agglomerans. According to the antimicrobial sensitivity test, antibiotics were changed to intraperitoneal ceftazidime plus oral ciprofloxacin for 14 days. The clinical outcome was favorable. No recurrence or sequelae was identified. Five months later, the patient was diagnosed with a leak of peritoneal fluid through the exit site of the Tenckhoff catheter, but no direct relation to the previous episode was found. The patient resumed treatment after four days with no further problems.

Case II

A 64-year-old Caucasian woman with CKD stage 5 due to Goodpasture syndrome was started on hemodialysis (HD). After three months, she chose to change to PD. Her hobby was gardening.

Fifteen months later, she complained of two days of abdominal pain, nausea, and vomiting. She was apyretic and hemodynamically stable. The ascitic fluid was cloudy and laboratory tests revealed PCC of 1.485/µL with 85% neutrophils. WCC was 7.8x10^9^ and CRP was 17.4 mg/dL. Microbiological cultures revealed *Pantoea* spp and Staphylococcus aureus in ascitic fluid. The patient was initially treated with empiric intraperitoneal cefazolin and ceftazidime. After cultures’ results, antibiotics were changed to intravenous vancomycin, gentamicin, and ciprofloxacin. At the same time, she was diagnosed with an abdominal hernia near the exit site of the peritoneal catheter and therefore she underwent surgical hernia correction and a new PD catheter was placed. She continued PD with no more complications.

Case III

A 45-year-old woman (Caucasian, oligophrenic patient) with CKD stage 5 of unknown etiology was started on PD. Her caregiver was a gardener. About one month after starting PD, she had an infection of the exit site due to methicillin-sensitive Staphylococcus aureus (MSSA), and three months later she was diagnosed with peritonitis also due to MSSA. The patient was successfully treated with intraperitoneal cefazolin.

Five months after starting PD, she presented with severe abdominal pain with 24 hours of evolution and no other symptoms. The patient was apyretic and hemodynamically stable with abdominal tenderness. There were no inflammatory signs peri-catheter, but the ascitic fluid was cloudy (PCC 1.600 cells/µL with 78% neutrophils). Blood samples revealed WCC of 7.0x10^9^ with 78% neutrophils and CRP of 0.6 mg/dL. The fluid culture was positive for *Pantoea* spp and Leclercia adecarboxylata. Furthermore, an abdominal wall hernia, on the subcutaneous tunnel of the PD catheter, was diagnosed. She was empirically treated with intraperitoneal cefazolin and ceftazidime, and later these antibiotics were changed to the intravenous route. PD catheter was removed, and the abdominal hernia was corrected. The patient was temporarily changed to HD and a new PD catheter was insert a month later. Currently, she is in a regular PD program.

In Table 1, we present the main features of our clinical cases.

**Table 1:** Summary of Pantoea PD peritonitis in our clinical cases ESKD, end-stage kidney disease; TNCC, total nucleated cell count; HT, hypertension; IP, intraperitoneal; IV, intravenous; PD, peritoneal dialysis

Clinical case, sex, age	ESKD cause	Dialysis vintage months	Epidemiological context	Effluent TNCC (/mm^3^)/neutrophils (%)	Microbiological agent	Antibiotic used/route
Case I, male, 63	Horseshoe kidney, calcium oxalate calculi, and HT	12	Gardening one week before. Abrasive wounds in the forearms	6,720/74	P. agglomerans	Ceftazidime and ciprofloxacin/IP
Case II, female, 64	Goodpasture syndrome	15	Hobby: gardening	1,485/85	Pantoea spp and Staphylococcus aureus	Vancomycin, gentamicin, and ciprofloxacin/IV
Case III, female, 45	Unknown etiology	1	PD helper was a gardener	1,600/78	Pantoea spp and Leclercia adecarboxylata	Cefazoline and ceftazidime/IV

## Discussion

Peritonitis is a serious PD complication and constitutes one of the major reasons for PD dropout. Meticulous adherence to sterile technique is very important to prevent contamination. For infection treatment with effective antibiotic coverage, a proper microbiological examination is crucial. According to the International Society for Peritoneal Dialysis (ISPD) guidelines, patients should be tested for peritoneal fluid cell count, gram stain, and culture for every peritonitis episode [7].

*Pantoea* spp infection is caused by plant thorn injuries, intravenous anesthetics catheters, contaminated parenteral nutrition, and subgingival sites with periodontal diseases [8].

This organism is perhaps underdiagnosed because it is not identified with the most common microbiology techniques. To our knowledge, only 11 cases of *Pantoea* spp PD peritonitis were published since the first report [6,8-17]. The slow growth of this organism in cultures, as described by Lim et al. [9], may account for some non-diagnosed cases and might only be possible when using blood culture media (BactAlert®). In our clinical cases, *Pantoea* was identified using the Vitek-2® automated system.

Cases reported here, like the previous ones, showed no apparent distinctive pattern of presentation. All patients were brought to medical attention with typical signs and symptoms of peritonitis: abdominal pain, cloudy peritoneal fluid, and elevated inflammatory parameters, in some cases exceptionally high [10,11]. All reported cases showed clean exit sites without peri-catheter inflammatory signs. In our first case, as well as in two previous reports [9,11], there was a description of arm wounds from gardening, before the occurrence of peritonitis, suggesting a hematogenous route of infection. In one case, there was a history of biting of the catheter (a two year-child under PD) with possible local contamination from commensal oral *Pantoea* [6], and in another case, a close relationship between the patient and a dog also suggested possible local contamination [12]. In another three cases [8,10,13,17], there was no significant event or suggested vehicle of infection; therefore, a possible translocation contamination route was admitted. A more recent published case [15] has been reported as the first case of Citrobacter younger and *Pantoea* agglomerans peritonitis in a PD patient. This 58-year-old diabetic patient had a recent history of osteomyelitis of the right toe to a methicillin-resistant Staphylococcus aureus. In two of our cases, there were two different species in the peritoneal fluid, which favors noncompliance with sterilization procedures as the cause of the infection.

Whenever facing a gram-negative bacteria in the direct examination with no bacteria isolation with the usual cultural media, and in the presence of risk factors (close contact with plants), one should consider *Pantoea* spp as the infectious bacteria. The incubation period of this bacteria is short, averaging from a few hours to two days between the inciting event and the initial clinical complaints.

The previously reported cases were treated with various antibiotics, initially according to the unit protocol and afterward changed according to the laboratory data. No multi-resistant *Pantoea* has ever been documented. The course of the antibiotic treatment ranged from 14 days to 3 weeks and the clinical outcome was consistently favorable, without recurrence of infection. Only one case documented a deleterious effect on the peritoneal membrane suggesting a link between *Pantoea* spp and an important loss of peritoneal permeability as a sequel of the peritonitis [13]. This organism is rarely fatal; nevertheless, there is in the literature, one case of death due to septic shock [14].

In Table 2, we review the published cases of PD peritonitis with *Pantoea* spp.

**Table 2:** Cases of Pantoea spp PD peritonitis described in the literature DM, diabetes; ESKD, end-stage kidney disease; PD, peritoneal dialysis; TNCC, total nucleated cell count; ST, successful treatment; HT, hypertension; IP, intraperitoneal; IV, intravenous; M, male; F, female; NA, not available; CAPD, continuous ambulatory peritoneal dialysis; APD, automated peritoneal dialysis

Case	Sex/age	ESKD cause	PD prescription/dialysis vintage (years)	Epidemiological context	Effluent TNCC (/mm^3^)/neutrophils (%)	Microbiological agent	Antibiotic used/route	Outcome
Lau et al. 2005 [6]	F/2	NA	NA	Teething on the catheter	NA	P. agglomerans	Cefotaxime and gentamicin/IP	ST and PD catheters replaced
Lim et al. 2006 [9]	M/49	Focal segmental glomerulosclerosis	CAPD/5	Rose-thorn injury	3,600/85	P. agglomerans	Ceftazidime and amikacin/IP	ST
Magnette et al. 2008 [10]	F/65	Left nephrectomy because of congenital hydronephrosis, HT, and DM	NA/2	Translocation from the GI tract	2,800/88	P. agglomerans	Cefazolin/IP and Ciprofloxacin/IV	ST
Ferrantino et al. 2008 [11]	M/51	NA	NA	Gardening 5 days before	3,600/74	P. agglomerans	Cefepime/IP	ST
Habhab and Blake 2008 [12]	F/52	Obstructive uropathy	NA	Close contact with a dog	10.8x10^9^/85	P. agglomerans	Ciprofloxacin/oral	ST
Borràs et al. 2009 [13]	F/56	Myeloma cast nephropathy and HT	CAPD/5	NA	320/88	P. agglomerans	Tobramycin and vancomycin/IV	ST
Kahveci et al. 2011 [14]	F/87	DM	APD/10	NA	3,400/NA	P. agglomerans	Sefuroksin and ciprofloxacin/IP. Later, imipenem/IV	Dead (septic shock) Patient refused PD catheter removal
Kazancioglu et al. 2014 [8]	F/63	HT	NA/1	Previous contact of the catheter with nonsterile surfaces	810/NA	P. agglomerans	Cefazolin and gentamicin/IP	ST
Chen et al. 2013 [15]	F/58	DM	NA/8	NA	2,190/NA	Citrobacter youngae and P. agglomerans	Levofloxacin/IV	ST
Choi et al. 2012 [16]	M/52	HT, liver cirrhosis	CAPD/NA	Diarrhea and episode of peritonitis 5 weeks before	5,940/91	P. agglomerans	Cefazolin and gentamicin/IP	ST
Sastre et al. 2017 [17]	M/83	Nephroangiosclerosis	CAPD/4	NA	560/µL/80	P. agglomerans	Cefazolin and tobramycin/IP	ST

## Conclusions

*Pantoea* might be an underdiagnosed agent of PD peritonitis. During PD patient education programs, it should be highlighted the care regarding proximity to plants and pets. *Pantoea* should be suspected when there is a compatible history increasing its risk, especially close contact with plants and perhaps animals and whenever facing the identification of a gram-negative bacilli without corresponding isolation in culture. The *Pantoea* spp peritonitis seems to respond well to antibiotics, administered at least for 14 days, and the prognosis is presumed to be good.
